# Diagnostic Dilemma of Differentiating Attention-Deficit/Hyperactivity Disorder (ADHD) From Mood Disorders and Other Common Psychiatric Illnesses in Substance Use Patient Population

**DOI:** 10.7759/cureus.37372

**Published:** 2023-04-10

**Authors:** Nazar Muhammad, Bhavani G Murugesan, Prakamya Singal, Angelica Antai, Lakshit Jain

**Affiliations:** 1 Psychiatry, Cornerstone Family Healthcare, New York, USA; 2 Psychiatry and Behavioral Sciences, State University of New York (SUNY) Upstate Medical University, Syracuse, USA; 3 Psychiatry, All India Institute of Medical Sciences, New Delhi, IND; 4 Radiology, University of Uyo Teaching Hospital, Uyo, NGA; 5 Psychiatry, University of Connecticut, Hartford, USA

**Keywords:** under diagnosed adhd, diagnostic dilemma, mood disorders, substance use disorder (sud), adult adhd

## Abstract

To raise awareness of attention-deficit/hyperactivity disorder (ADHD) as an underdiagnosed, undertreated disorder in adult patients with comorbid substance use disorder (SUD) who are misdiagnosed with other common psychiatric illnesses and to reduce fear and hesitancy in prescribing stimulants as treatment in such a patient population. ADHD diagnosis is easier in the child and adolescent population than the adults due to comorbidities of other psychiatric illnesses and SUD. However, diagnosing ADHD appropriately in an increasing number of adult patients presents challenges. Even if they get diagnosed appropriately, the stigma of substance use disorder holds the providers prescribing stimulant medications for such patient populations due to the high comorbidity of ADHD with SUD. Accurate diagnosis of ADHD in adults is a worthwhile endeavor as this diagnosis is comorbidly present in many mood and substance use disorders patients. Treating ADHD in this population can improve clinical symptoms and overall quality of life.

## Introduction

Adults with substance use disorders (SUD) and attention-deficit/hyperactivity disorder (ADHD) are increasingly presenting in clinical practice [1]. It carries significant impediments in academic, occupational, social, and intrapersonal domains necessitating treatment. Converging data strongly supports a neurobiological and genetic basis for attention-deficit/hyperactivity disorder (ADHD) with catecholaminergic dysfunction as a central finding. Consideration of all aspects of an individual's life is needed to diagnose and treat ADHD [2]. Symptoms of ADHD included subsyndromal mood swings, low self-esteem, and low energy and motivation levels. They decreased cognitive abilities, including impaired focus and concentration, often overlapping with symptoms of common mood disorders such as cyclothymia, persistent depressive disorder, bipolar disorder, and rarely psychotic disorder. Consequently, adults with ADHD are misdiagnosed with mood disorders. The use of illegal street drugs, the symptoms of which resemble features of both ADHD and mood disorders, further complicates arriving at a correct diagnosis. The Diagnostic and Statistical Manual of Mental Illnesses (DSM)-5 uses two criteria for diagnosing ADHD in adults and children. Inattention and hyperactivity-impulsivity for more than six months, with clear evidence that the symptoms interfere with social or occupational functioning in more than two settings, are required to diagnose. In adults and children, for diagnosis of ADHD, several symptoms of inattention or hyperactivity-impulsivity must have been present before 12 years of age, a shift from the criterion set in DSM-4. Diagnosis can be made in an adult if five or more symptoms of either of the two measures are present. In a child less than 17 years of age, six or more signs are required for a diagnosis of ADHD. In addition, the symptoms must be excessive compared with a child's average developmental level. Presentation of ADHD in adults and children can be predominantly of inattention or hyperactivity-impulsivity type or a combination of the two criteria. 

## Case presentation

Mr. X was self-referred to our outpatient clinic for medication-assisted treatment for opioid use disorder and adjustment of his psychotropic medications regimen. He is a homeless 28-year-old Caucasian male living in a shelter and receiving support from the Department of Social Services (DSS). This patient had been previously diagnosed with bipolar disorder, high-functioning autism spectrum disorder, post-traumatic stress disorder (PTSD), and opioid use disorder (on Suboxone® R).

At intake, he endorsed craving opioids and compulsive behavior to obtain opioids. Mr. X's daily medications included haloperidol 10 mg twice daily, bupropion 300 mg extended-release (XL) daily, and divalproex sodium delayed-release (DR) 500 mg in the morning and 1000 mg at bedtime for mood swings. He noted none or minimal improvement in these symptoms despite medication compliance for more than three months. The divalproex sodium level was obtained and within the therapeutic range. All other routine labs were normal. During history taking, he verbalized subsyndromal mood swings, including feeling sad with low self-esteem and low levels of energy and motivation most of the time. He stated that medications, including Suboxone®, helped his cravings and compulsive opioid-seeking behavior but did not help control his mood swings despite of using Suboxone® for more than six months. Though Mr. X carried the diagnosis of high-functioning autism spectrum disorder, he did not have behavioral problems and denied any anxiety disorder symptoms related to the same.

He had two prior psychiatric hospitalizations in the context of worsening depression and elevated mood. His hospitalization had resulted from an inability to focus, concentrate and live his life independently, which worsened his mood symptoms. Various serotonin reuptake inhibitors (SSRI) and serotonin and norepinephrine reuptake inhibitors (SNRI) taken with the above medications did not significantly improve. He lost his job due to his inability to focus and concentrate. As a result, he could not financially support himself and was kicked out by his family members. He had a history of smoking marijuana, around four to five puffs daily, and was attending contingency management group therapy to relieve his potential symptoms of underlying ADHD and mood disorder.

Aftercare was established, and Mr. X's symptoms were monitored for about three months. He was initially tried on oxcarbazepine for two months due to minimal clinical response. A decision to taper all his psychotropic medications (bupropion, oxcarbazepine, and haloperidol) was made. His symptoms showed no improvement, and the patient was monitored for potential worsening psychosis and mood disorder. Eventually, a decision was made to start Vyvanse® (lisdexamfetamine) 20 mg daily, empirically for diagnosis and to relieve his symptoms as the patient was resistant to traditional psychotropic medications and was manifesting subsyndromal mood swings, including low self-esteem, low level-of energy, motivation and poor impulse control. He was followed for four week on Vyvanse® 20 mg. 

After starting Vyvanse®, Mr. X's mood improved, resulting in him seeking an employment. He verbalized minimal mood swings. Slowly, the Vyvanse® dose was titrated to 40 mg daily, which further improved the remainder of Mr. X's symptoms. He started doing better emotionally and showed improvement in his interpersonal, functional, and occupational life. 

Based on his history, clinical presentation, nonresponse to mood-stabilizing drugs, and improvement of symptoms on Vyvanse®, a diagnosis of ADHD was made, and the prior diagnosis of bipolar disorder was removed. He was continued on Vyvanse® and Suboxone® for ADHD and Opioid use disorder.

## Discussion

Adults with concomitant SUD and ADHD are a common presentation in clinical practice. Research shows a connection between the two conditions, with over 15% of young adults with ADHD having comorbid SUD [3]. ADHD is a clinically heterogeneous neurodevelopmental syndrome characterized by inattentiveness, hyperactivity, and high impulsivity. The condition affects numerous aspects of life and is associated with sleep disorders, unemployment, poor interactions, career failure, and criminality [4,5]. People with ADHD are more likely to develop SUD than the general population [6,7]. Research reveals significant links between ADHD and other comorbid psychiatric disorders such as anxiety, depression, bipolar disorder [8], learning disabilities, and substance use disorder, including methamphetamine and nicotine use disorder [7]. Over 75% of adults with ADHD have at least one other psychiatric diagnosis [8,9]. Symptoms can also vary widely from person to person [1], thus making ADHD in adults challenging to diagnose. Some of the reasons ADHD is often missed as a diagnosis include a lack of awareness among physicians, stigma, and self-medication [10].

Aspects that caused a misdiagnosis in Mr. X’s case include changes in symptoms due to age and overlapping symptoms with other comorbidities. As such, patients with ADHD are often underdiagnosed and undertreated [8]. The high comorbidity between ADHD and SUD and the resulting social risks has prompted some investigators to explore the possibility of underlying neurobiological substrates. Since the symptoms of ADHD closely resemble mood disorders, a patient with other substance use disorders is a challenge to diagnose accurately. Even with the correct diagnosis, providers hesitate to prescribe stimulants in substance-use populations. Neuroimaging studies of ADHD have revealed similarities with SUD in both positron emission tomography (PET) and functional magnetic resonance imaging (fMRI) studies, particularly between patients with ADHD and those with addiction-related cravings [7].

Considering Mr. X’s case, one must also consider the role of a hedonic tone in his clinical presentation. Sternat and Katzman have argued that low hedonic tone is a common denominator in ADHD, SUD, and depression and that the primary neural pathway implicated in emotional affect is the limbic-cortical-striatal-pallidal-thalamic circuits. Dysfunction in various components of this circuitry has also been implicated in developing the aforementioned related psychopathologies and the potential for poor response to selective serotonin reuptake inhibitors (SSRIs) [11]. Our index case had a poor response to oral haloperidol, sodium divalproex, and SSRIs which likely contributed to a negative impact on the hedonic tone if one considers a neuroleptic-induced deficit syndrome hypothesis in the context of bipolar disorder [11].

Stigma has been explored extensively in the context of psychiatric disorders. Stigma is a crucial determinant in help-seeking, treatment outcomes, and quality of life of individuals with psychiatric conditions [12]. Due to the stigma associated with ADHD, individuals may hesitate to report actual symptoms to physicians or may downplay them, leading to underdiagnosis or misdiagnosis [13]. The main categories of stigma include self-stigma, structural stigma, and public stigma. Self-stigma entails having negative feelings about oneself, mainly emerging from past experiences and perceptions about the reaction of others toward the psychiatric disorder [14]. Structural stigma is associated with institutional policies and practices that affect people with psychiatric problems [12], for example, how mental health is reported on various media platforms. Even if the patient reports such symptoms, comorbid substance use and the resemblance of ADHD symptoms with other psychiatric illnesses make it even more difficult to diagnose it accurately. Public stigma comprises a set of negative conceptions about people with psychiatric disorders, causing them to be feared, avoided, and discriminated against. Psychological disorders may be associated with dangerousness, impulsiveness, and wrongdoing [14]. Such individuals may feel threatened by the public, and the threat level varies across different conditions [12]. Stigmatization associated with criminality in ADHD patients has been widely reported. People with SUD also face public stigma, which leads to adverse outcomes, including an unwillingness to disclose symptoms or seek treatment. 

The disorder is linked with a 30-140% increase in morbidity and 85-110% in mortality rates [15]. Adults with ADHD frequently struggle in various aspects of their lives because they rarely receive the right diagnosis and treatment they require [16]. Sibley found that ADHD diagnostic criteria often consisted of school-aged indications, thus presenting clinicians with suboptimal diagnostic guidelines for adults [17]. If left untreated, ADHD is reported to cause a decline in the quality of life, including the use of illegal drugs [15]. Over half of the individuals diagnosed with ADHD have substance use disorder [18]. Therefore, pharmacotherapy is essential in ADHD management, as is psychotherapy. Adults with comorbid ADHD and SUD find a high success rate with psychotherapy, a strategy fundamental in ADHD management. 

In the presented case, Mr. X had been diagnosed with opioid use disorder and had two previous hospitalizations due to depression and elevated mood. He also had a history of smoking marijuana. Substance use often mimics ADHD symptoms. For example, uncontrolled marijuana use is linked with impairments in cognitive functioning, memory, and attention [17]. Mr. X portrayed a wide range of symptoms, including ADHD and SUD. Researchers agree that aspects that cause drug abuse among ADHD patients include self-medication, behavioral disinhibition, comorbidity, and sensitization. A Danish follow-up study revealed that individuals diagnosed with ADHD are likely to have developed SUD by the age of 31 years [18]. People with ADHD may also experience contraindicative effects while on certain drugs. For example, one study found that amphetamine enhances focus and concentration in ADHD patients comparable to “a speed buzz” [18]. A comparable effect is experienced with nicotine, similar to psychostimulant medications. 

For each specific case, the guidelines for ADHD management and treatment may vary. The context in which the treatment is being administered and the country’s guidelines are crucial aspects to consider in ADHD management. The International Collaboration of ADHD and Substance Use (ICASA) recommends integrating SUD and ADHD treatment early enough. The first line of treatment includes the use of psychostimulants, mostly methylphenidate and amphetamine formulations [15,18]. Response to stimulant medications differs by the patient, with some responding to methylphenidate and others to amphetamine. International standards on treating ADHD highlight the need for cognitive behavioral therapy as a viable nonpharmacological treatment in dual diagnosis for SUD and ADHD [19]. The National Institute for Health and Clinical Excellence (NICE) recommends implementing a treatment program that includes detoxification, incentive programs, behavioral family therapy, and cognitive behavioral therapy [20]. While most interventions address the dependency phase of use, practitioners need to focus on early detection and intervention.

## Conclusions

ADHD, SUD, and other common psychiatric illnesses occur comorbidly in adults. Symptoms of ADHD closely resemble mood disorders, and in a patient with concomitant substance use disorder, an accurate diagnosis of ADHD becomes a challenge. Physicians often hesitate to prescribe stimulants in such illicit drug-use patient populations as stimulants carry the risks of addiction and abuse. Though the clinical picture of our patient was unclear, starting and monitoring stimulants diagnostically changed this patient's life. This case represents another example where prescribing necessary medications (e.g., a stimulant in Mr. X's case) without any delay improved clinical symptoms and the quality of life of a patient. It provides another example where hesitancy in prescribing appropriate medications should be challenged. Understanding different ADHD and mechanisms of action of medication options allows clinicians to customize treatment to each patient's needs and attain better outcomes. Ultimately, the quality of life of a person with comorbid ADHD and SUD improves with the proper diagnosis and treatment.
